# Development and validation of a RAD-Seq target-capture based genotyping assay for routine application in advanced black tiger shrimp (*Penaeus monodon*) breeding programs

**DOI:** 10.1186/s12864-020-06960-w

**Published:** 2020-08-05

**Authors:** Jarrod L. Guppy, David B. Jones, Shannon R. Kjeldsen, Agnes Le Port, Mehar S. Khatkar, Nicholas M. Wade, Melony J. Sellars, Eike J. Steinig, Herman W. Raadsma, Dean R. Jerry, Kyall R. Zenger

**Affiliations:** 1 Australian Research Council Industrial Transformation Research Hub for Advanced Prawn Breeding, James Cook University, Townsville, QLD 4811 Australia; 2 Centre for Sustainable Tropical Fisheries and Aquaculture, College of Science and Engineering, James Cook University, Townsville, QLD 4811 Australia; 3 Sydney School of Veterinary Science, Faculty of Science, The University of Sydney, Camden, NSW 2570 Australia; 4 CSIRO Agriculture & Food, Integrated Sustainable Aquaculture Production Program, Queensland Bioscience Precinct, St Lucia, QLD 4067 Australia; 5 Present Address: Genics Pty Ltd, Research Road, St Lucia, QLD 4011 Australia; 6 Australian Institute of Tropical Health and Medicine, James Cook University, Townsville, QLD 4811 Australia; 7 Tropical Futures Institute, James Cook University, Singapore, Singapore

**Keywords:** Genotype by sequencing, Advanced breeding, Black Tiger shrimp, *Penaeus monodon*, Diversity arrays technology, Aquaculture

## Abstract

**Background:**

The development of genome-wide genotyping resources has provided terrestrial livestock and crop industries with the unique ability to accurately assess genomic relationships between individuals, uncover the genetic architecture of commercial traits, as well as identify superior individuals for selection based on their specific genetic profile. Utilising recent advancements in *de-novo* genome-wide genotyping technologies, it is now possible to provide aquaculture industries with these same important genotyping resources, even in the absence of existing genome assemblies. Here, we present the development of a genome-wide SNP assay for the Black Tiger shrimp (*Penaeus monodon*) through utilisation of a reduced-representation whole-genome genotyping approach (DArTseq).

**Results:**

Based on a single reduced-representation library, 31,262 polymorphic SNPs were identified across 650 individuals obtained from Australian wild stocks and commercial aquaculture populations. After filtering to remove SNPs with low read depth, low MAF, low call rate, deviation from HWE, and non-Mendelian inheritance, 7542 high-quality SNPs were retained. From these, 4236 high-quality genome-wide loci were selected for baits-probe development and 4194 SNPs were included within a finalized target-capture genotype-by-sequence assay (DArTcap). This assay was designed for routine and cost effective commercial application in large scale breeding programs, and demonstrates higher confidence in genotype calls through increased call rate (from 80.2 ± 14.7 to 93.0% ± 3.5%), increased read depth (from 20.4 ± 15.6 to 80.0 ± 88.7), as well as a 3-fold reduction in cost over traditional genotype-by-sequencing approaches.

**Conclusion:**

Importantly, this assay equips the *P. monodon* industry with the ability to simultaneously assign parentage of communally reared animals, undertake genomic relationship analysis, manage mate pairings between cryptic family lines, as well as undertake advance studies of genome and trait architecture. Critically this assay can be cost effectively applied as *P. monodon* breeding programs transition to undertaking genomic selection.

## Background

Genotype-by-sequencing (GBS) has quickly been recognised as a highly versatile and cost-effective approach to rapidly generate genome-wide marker data for an emerging aquaculture species, or those otherwise lacking existing SNP-based genomic resources [1]. In particular, since publication of the first Restriction-site Associated DNA Sequencing (RAD-Seq) protocol by Miller [2], a number of innovative developments of RAD-Seq protocols have been published. These include RAD [3], DArTSeq [4], ddRAD [5], 2bRAD [6], ezRAD [7] and 3RAD [8], all of which have seen dramatic improvements in the ease of generating reliable, repeatable and low-cost genotype data through GBS methods across a plethora of species (reviewed by [9]). As such RAD-Seq protocols have been increasingly applied within a number of aquaculture and fisheries fields of research (reviewed by [1, 10]).

Across the range of RAD-Seq methods now available, each protocol contains its own subtle differences in methods undertaken throughout the sample-to-sequencing process, which have been reviewed in detail by Puritz [11], Andrews [9] and Robledo [1]. Importantly however, all commonly utilized methods (e.g. RAD, [3]; ddRAD, [5]; DArTSeq, [4]) share the fundamental approach of sequencing only a reduced representation of each individuals’ genome. Specifically, by undertaking genomic complexity reduction steps (i.e. restriction enzyme digestion and/or fragment size selection) next-generation sequencing efforts can be focused more efficiently by consistently sequencing specific regions across each individuals’ genome [9].

In an effort to further ensure consistent sequencing of the same genomic regions, a number of RAD-Seq methods have been paired with ‘targeted sequence capture’ protocols to enrich desired sequences (known as ‘RAD-tags’) in the sequencing libraries before final preparation for high-throughput next-generation sequencing [12]. Specifically, these approaches [e.g. Rapture, [13]; hyRAD, [14]; DArTCap, unpublished modifications of Sansaloni [4]] utilize bead-based hybridization (e.g. DYNAbeads®) or capture baits (e.g. MYbaits®) to exclusively select RAD-tags from DNA samples that have already undergone previous traditional complexity reduction steps. This second stage of library refinement is referred to as ‘enrichment’ [12].

By following this two-stage library preparation approach, it is possible to further improve the consistency of genotype data compared to traditional RAD methods in two ways. Firstly, it is possible to obtain higher sequence read coverage of a refined set of loci which improves confidence in genotype calls [13]. Secondly, through multiplexing more samples within a fixed allocation of sequencing effort, it is possible to substantially reduce the genotyping cost per individual [9, 13, 15, 16]. By leveraging these additional strengths of ‘RAD-Seq target-capture’ hybrid protocols, genotyping strategies (i.e. adjusting optimal sequence depth and/or multiplexing a higher number of individuals) can be tailored to efficiently fulfil the intended uses of genotype data in aquaculture (i.e. tracing pedigree, allocating mate-pairings and determining family contributions). Furthermore, when ‘RAD-seq target capture’ genotyping is coupled with the collection of large phenotypic datasets, a plethora of advanced applications can be achieved. The calculation of genomic relationship matrices (GRM), genomic estimated breeding values (GEBV); discovery of selection signatures, implementation of genomic selection (GS), genome-wide association studies (GWAS), quantitative trait loci mapping (QTL), and genetic marker imputation are now common place in the study and management of most terrestrial livestock species.

Black tiger shrimp (*Penaeus monodon*) are an aquaculture species of significant value (~$USD 4.5 billion [17];), however, the industry has been troubled by inconsistent seedstock quality and numerous devastating disease outbreaks. While producing a current global production of 713,318 metric tonnes per annum [17], the industry stands to benefit greatly from developing genetic tools to manage existing breeding programs more effectively, and furthermore, facilitate a transition to genomic based breeding programs (i.e. genomic selection [18–21];). While efforts have been undertaken to develop a range of genomic markers (e.g. microsatellites [22–24]), AFLPs [25], low-density (59–122) SNPs [26]), these marker panels lack the power for applications required in advanced breeding programs particularly when considering complex traits like pathogen resistance or tolerance [20, 26, 27]. The only existing medium density SNP-based marker panel (6000 SNPs) produced for black tiger shrimp was developed upon the high-cost Illumina iSelect array platform, and as we are aware, has yet to be made accessible for commercial use [28]. To date, no genotyping assay has been produced for black tiger shrimp that can feasibly be applied in routine high-volume applications or to service the industries desire to progress towards advanced selective breeding programs.

Here, the processes and rational underlying the development of a hybrid ‘RAD-Seq target-capture’ GBS assay for application within a black tiger shrimp industrial aquaculture setting are detailed. We demonstrate the versatility of this assay through traditional parentage assignment of animals reared under communal commercial conditions (i.e. pedigree unknown), as well as the utility of this assay in GRM calculations which set the foundations for accurate estimations of GEBVs. The ability to generate accurate GEBVs is integral for the establishment of genomic selection programs in black tiger shrimp.

## Results

### SNP discovery, quality assessment and selection

After processing raw sequencing data from a DArTseq GBS library of 650 individuals (ten individuals were excluded during library preparation), a dataset of 24,683 RAD-tags containing 31,262 SNP markers (31 K SNP dataset), was returned. An average of 1.37 SNPs ±0.6 SD were present in each RAD-tag, with a maximum of six SNPs observed in a single RAD-tag (19,288 tags with one SNP, 4300 tags with two SNPs, 915 tags with three SNPs, 155 tags with four SNP, 22 tags with five SNP, three tags with six SNPs). For this raw 31 K SNP dataset, the average genotype call rate was 0.86 ± 0.14 SD and the average MAF was 0.11 ± 0.15 SD.

An average of ~ 2.5 million reads were allocated to each individual in library preparation and sequencing; however, after removal of low quality sequences, monomorphic loci, and SilicoDArT markers (presence/absence variants), an average of 459,987 ± 88,493 SD reads were associated with each individual (366,609,683 total reads over 650 individuals). The average read depth over all non-missing genotype calls was 17.0 ± 18.3 SD, however, 27.0% of non-missing genotype calls had 5 or less reads associated.

**Fig. 1:**
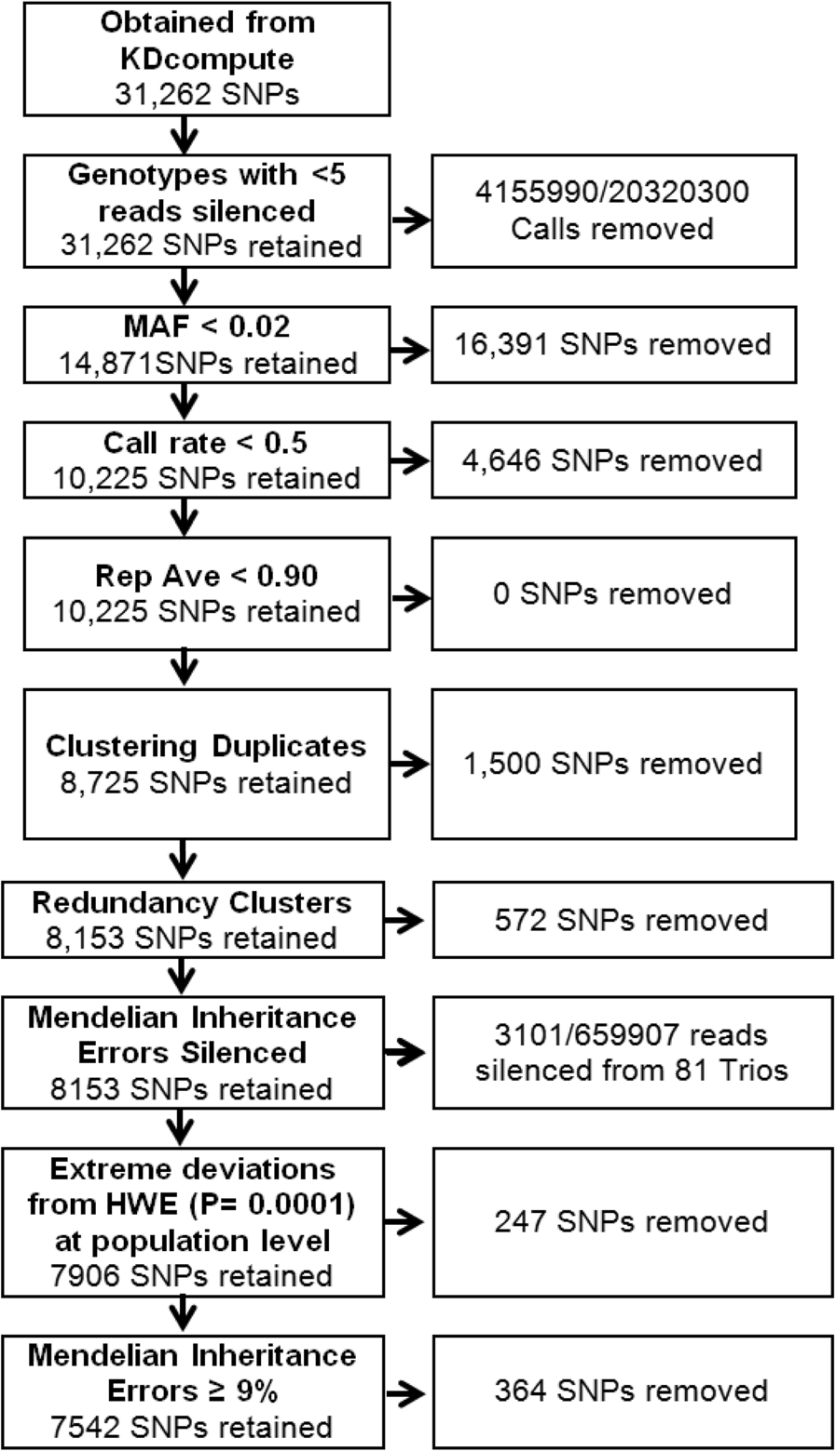
SNP qualtity control pipeline for development of *Peneaus monodon* genotyping assay and the number of SNPs retained after each step of filtering

To ensure only the highest quality markers were available for baits probe selection and assay development, a series of SNP filtering thresholds were implemented to remove individual genotype calls with low confidence, remove less informative markers and remove erroneous data (Fig. 1). Filtering for minimum read depth (< 5) removed 20.4% of the non-missing genotype calls (4,155,990), increasing the overall missing-ness of the 31 K dataset from 14 to 33%. Subsequently filtering for minimum minor allele frequency of ≥0.02 removed 16,391 SNPs. A further 4646 SNPs were removed due to having a minimum call rate equal to or below 0.5; no SNPs were discarded due to having a repeatability score of less than 0.9 due to pre-filtering on repeatability before data was provided. For SNPs that were derived from identical clones (100% rad-tag sequence similarity), the SNP with the highest MAF was retained from each clone resulting in the removal of 1500 SNPs. Similarly, redundancy clustering of clone sequences at 95% sequence similarity removed a further 572 SNPs derived from highly similar clones. Of the markers retained, tests of conformity to Mendelian inheritance patterns and HWE were conducted. A total of 364 SNPs were removed due to MI errors (> 9%) and 247 SNPs were removed due to HWE deviations. Finally, 3101 genotype calls identified as MI errors across the remaining SNPs were silenced (Fig. 1).

In total 23,720 SNPs were removed from the 31 K dataset, leaving 7542 high quality SNPs for further ranking and final SNP selection. After filtering, the average MAF increased from 0.11 ± 0.15 to 0.22 ± 0.15. While the average per genotype call rate decreased from 0.86 ± 0.14 to 0.84 ± 0.14, the average read depth per genotype call increased from 17.0 ± 18.2 SD to 30.7 ± 27.9 SD. Furthermore, the rate of average Mendelian inheritance errors detected across SNPs decreased from 3.2 to 1.4% after filtering.

#### SNP number and GRM analysis

**Fig. 2:**
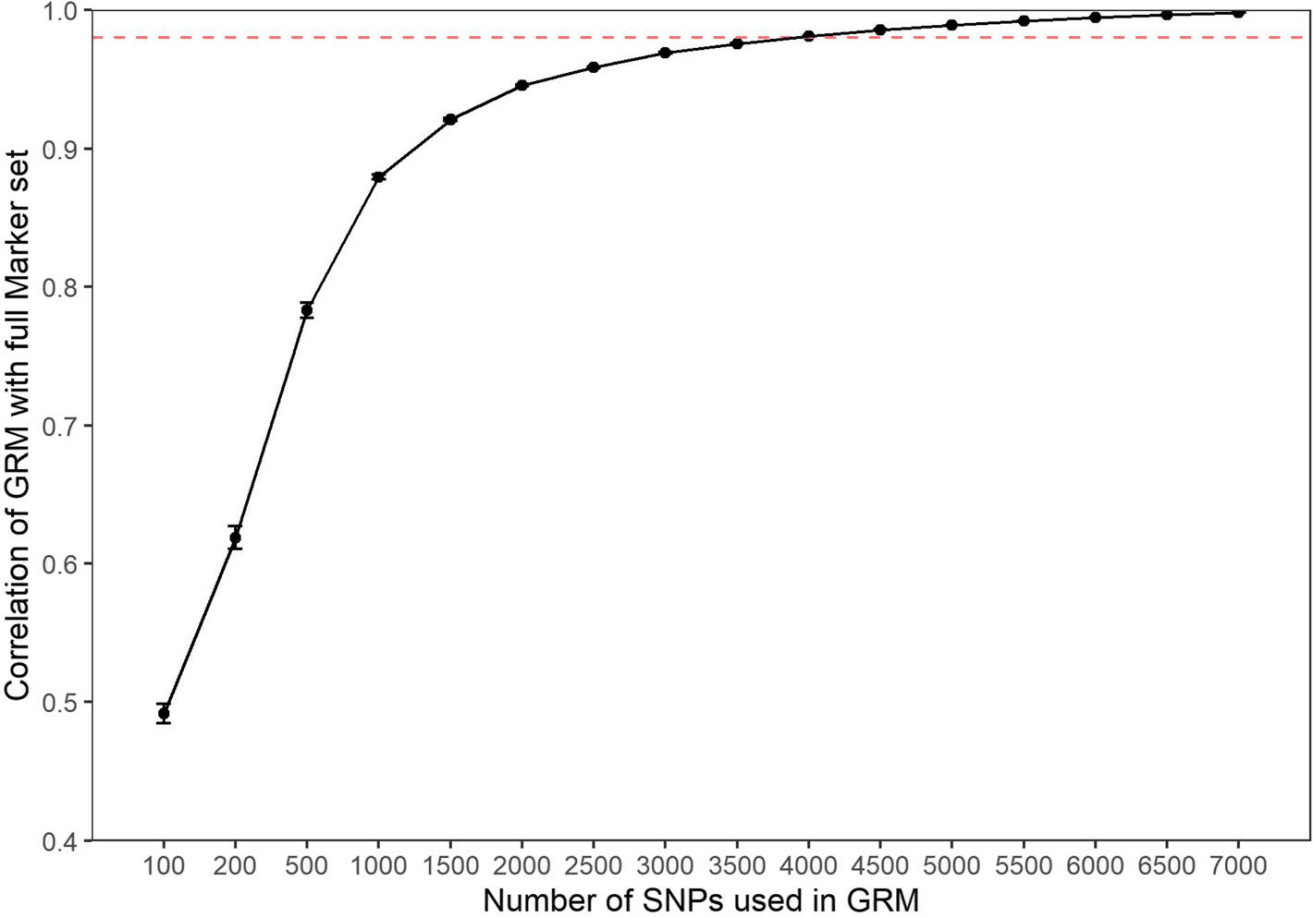
Correlations between GRMs estimated from randomly selected subsets of *n* marker density (G_*ni*_) and the complete pool of available markers (7542 SNPs). Average correlations and error (SE) of each *n* derived from 50 replicated GRM estimates. Desired correlation of > 0.98 indicated by the dashed line

To determine the number of markers required for accurate genomic relationship calculations and selection of the density of the DArTcap panel, a number of marker subsets were modelled. An increasingly consistent GRM estimate was achieved by including progressively more random markers from 100 to 1000 markers (Fig. 2). With 1000 markers a correlation of 0.95 was consistently achieved when compared to the full 75,442 marker set (Fig. 2). Increasing the number of markers from 1000 to 4000 further increased the correlation between marker sets, with the correlation exceeding the desired minimum cut off for future assay applications of 0.98 at 4000 markers (Fig. 2).

**Fig. 3:**
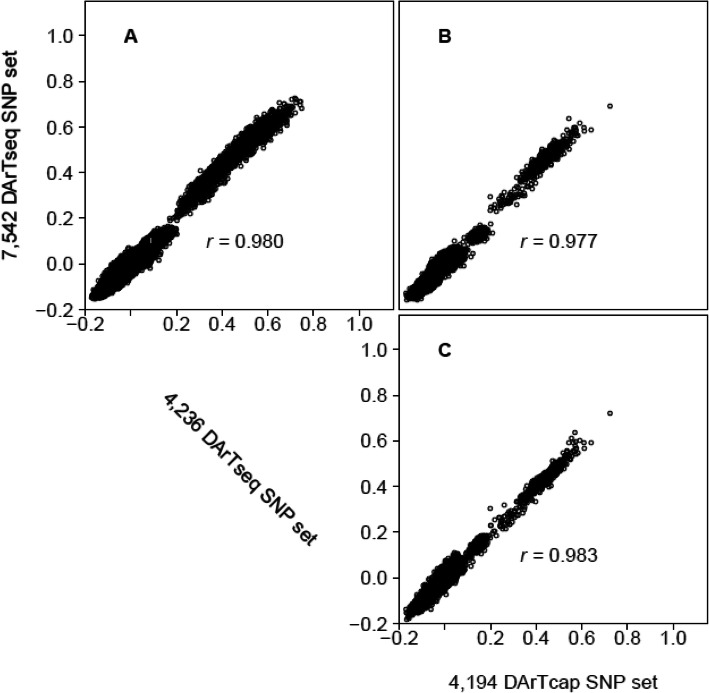
Comparison of genomic relationship values calculated from the full 7542 SNP set, the selected 4236 SNP set provided for DArTCap probe synthesis, and the final set of 4194 DArTcap SNPs. GRMs were calculated with all common individuals available between datasets including; 650 individuals (**a**), 195 individuals (**b**) and 195 individuals (**c**) respectively

Since GRM correlations indicated an optimal assay size of 4000 SNPs, a QC score was assigned to the set of 7542 high quality DArTseq markers allowing the prioritisation of 4236 SNPs for DArTcap probe systhesis. For this selected subset of markers, the average call rate was 80.2% ± 14.7%, MAF was 0.35 ± 0.28 and the average read depth per non-missing genotype call was 20.4 ± 15.6. Furthermore, genomic relationship values calculated with the 4236 markers showed high correlation (*r*^*2*^ = 0.987) to the full 7542 marker panel (Fig. 3a). A number of pairwise relationships were estimated to be negative. These values are a result of the distinct underlying population structure between individuals from East Australina Coast populations and those from Northern Territory populations [29].

#### Linkage disequilibrium

Limited evidence of substantial linkage disequilibrium was observed with the DArTseq datasets. A total of 4 marker pairs were found to be linked (*r*^*2*^ > 0.80) in the 7542 SNP dataset when considering all available individual genotypes, while only two of these marker pairs persisted within the selected 4236 SNPs (*r*^*2*^ values of 0.96 and 0.85). When assessing genotypes of the Northern Territory individuals only (Tiwi Island, Joseph Bonaparte Gulf, Gulf of Carpentaria) from the 7542 SNP dataset, there were three marker pairs with *r*^*2*^ values greater than 0.8. Likewise, for East Australian Coast individuals only (Bramston Beach, Townsville, Etty Bay), there were 13 *r*^*2*^ values greater than 0.80, however, only one of these was also observed in the Northern Territory population. Using only Northern Territory individuals from the 4236 SNP dataset, there was one marker pair identified to be in LD (*r*^*2*^ > 0.80). Likewise, when considering genotypes of East Australian Coast individuals, there were 12 *r*^*2*^ values greater than 0.80, with one pair shared with those observed in the Northern Territory population.

### DArTcap assay validation

A number of samples resubmitted for genotyping did not pass sample digestion QC, and were excluded from sequencing (Table 1). This was most prominently observed in the initial set of 2nd generation farm stock (44 of 90 submitted), and was identified to be due to DNA degradation during storage. Across the remaining 462 individuals, 21 samples from across various populations failed QC and were excluded (Table 1). Processing of raw sequencing data obtained from a DArTcap target-capture library including a total of 485 individuals produced a dataset of 15,880 RAD-tags containing a total of 26,751 SNP markers (raw DARTcap dataset). An average of 1.7 SNPs ±1.0 SD were present in each RAD-tag, with a maximum of 18 SNPs observed in a single RAD-tag (9110 tags with 1 SNP, 4142 tags with 2 SNPs, 1731 tags with 3 SNPs, and 897 with > 3 SNPs). For the raw DArTcap dataset, the average individual call rate was 72.0% ± 6.8% SD, average genotype call rate was 72.0% ± 25.17% SD, and average MAF was 0.11 ± 0.13 SD. Approximately 600,000–700,000 reads were allocated to each individual in library preparation and sequencing, and after removal of low quality sequences, an average of 670,610 ± 185,993 reads were associated with each individual (328,598,913 total). The average read depth per genotype call was 34.8 ± 61.8 SD, however 32.0% of non-missing genotype calls had 5 or less reads associated.

**Table 1:** Individuals genotyped with DArTseq and DArTcap

Population	Region	DArTSeq		DArTCap	
		# submitted	# passing library preparation	# submitted	# passing library preparation
Townsville	East Coast, Australia	22	22	10	10
Etty Bay	East Coast, Australia	50	50	15	7
Bramston Beach	East Coast, Australia	60	60	12	9
Gulf of Carpentaria	Northern Territory, Australia	42	35	14	14
Tiwi Island	Northern Territory, Australia	56	56	10	10
Joseph Bonaparte Gulf	Northern Territory, Australia	34	34	13	13
Nickol Bay	Western Australia, Australia	–	–	19	18
1st Generation	Farm Stock	165	162	87	86
2nd Generation Set 1	Farm Stock	231	231	90	46
2nd Generation Set 2	Farm Stock	–	–	282	272
Total		660	650	552	485

Within the raw DArTcap dataset, 4194 (99.0%) of the selected 4236 panel were observed, with only 52 baits probes failing to capture the desired rad-tag sequences. For the 4194 DArTcap dataset, the average individual call rate was 93.0% ± 3.5% SD, and average MAF was 0.23 ± 0.15 SD. An average of 312,343 ± 83,235 reads were associated with each individual (153,048,102 total). An average of 46.8% ± 1.6% of the total DArTcap sequencing effort was successful in obtaining target sequences per individual. The average read depth per non-missing genotype call was 80.0 ± 88.7 SD, while 5.78% of non-missing genotype calls had 5 or less reads associated.

### DArTcap assay utilisation

#### Parentage assignment

The results of parentage assignment success using Colony are presented in Table 2. At a conservative genotyping error rate of 10%, parentage assignment across the three sets of markers (7542 DArTseq, 4236 DArTseq, 4194 DArTcap) were essentially identical, where only one individual (from 72 known parent-progeny relationships) had a single parent incorrectly left unassigned (false exclusion) when using the 4194 DArTcap marker set (1.39% assignment error). The occurrence of false exclusions increased to 4.2 and 8.3% for the DArTcap marker set when using genotyping error rates of 5 and 1% respectively. The rate of false exclusions (2.3%) remained consistent irrespective of the error rate used in Colony for both the 7542 and 4236 DArTseq marker sets; however, the individual false exclusions did vary between either exclusion of the mother or father for some progeny.

**Table 2:** Success rate of parentage assignment analysis using three SNP marker sets (7542 DArTseq, 4236 DArTseq, 4194 DArTcap) and three genotyping error rates that range from strict to conservative (0.01, 0.05 and 0.1) in Colony [30]. There were no untrue parent assignments for any dataset at any error rate

SNP dataset	Success rate of parentage assignment at different genotyping error rates		
	0.01	0.05	0.10
7542 DArTseq	97.2%	97.2%	97.2%
4236 DArTseq	97.2%	97.2%	97.2%
4194 DArTcap	91.8%	95.8%	98.6%

Across all analyses, progeny containing higher missing data (> 10%) accounted for the majority of false parent exclusions. Furthermore, irrespective of the markers used in the analysis, there was a single individual where at least one of the two true parents could not be assigned consistently. For this individual, across analyses the unassigned parent was not consistent with either both parents unassigned, only the mother unassigned, or only the father unassigned. There were no untrue parent assignments (false assignments) observed across any of the assignment analyses, with all putative parents correctly excluded where the true parent/s were absent from the dataset tested.

#### Genomic relationship calculations

Genomic relationship values calculated with the 4194 DArTcap markers showed high correlation to the 7542 DArTseq marker set (*r*^*2*^ = 0.98; Fig. 3b) and the 4236 DArTseq marker panel ((*r*^*2*^ = 0.98; Fig. 3c), importantly indicating high concordance between the genotypes obtained for the 195 samples that were genotyped through the two GBS approaches. As with the comparision of DArTseq 7542 and 4236 marker panels (see above) a number of pairwise relationships were less than 0 (no relationship). This is likely to be due to the structured nature of the wild samples included in the dataset [29, 31, 32].

**Fig. 4:**
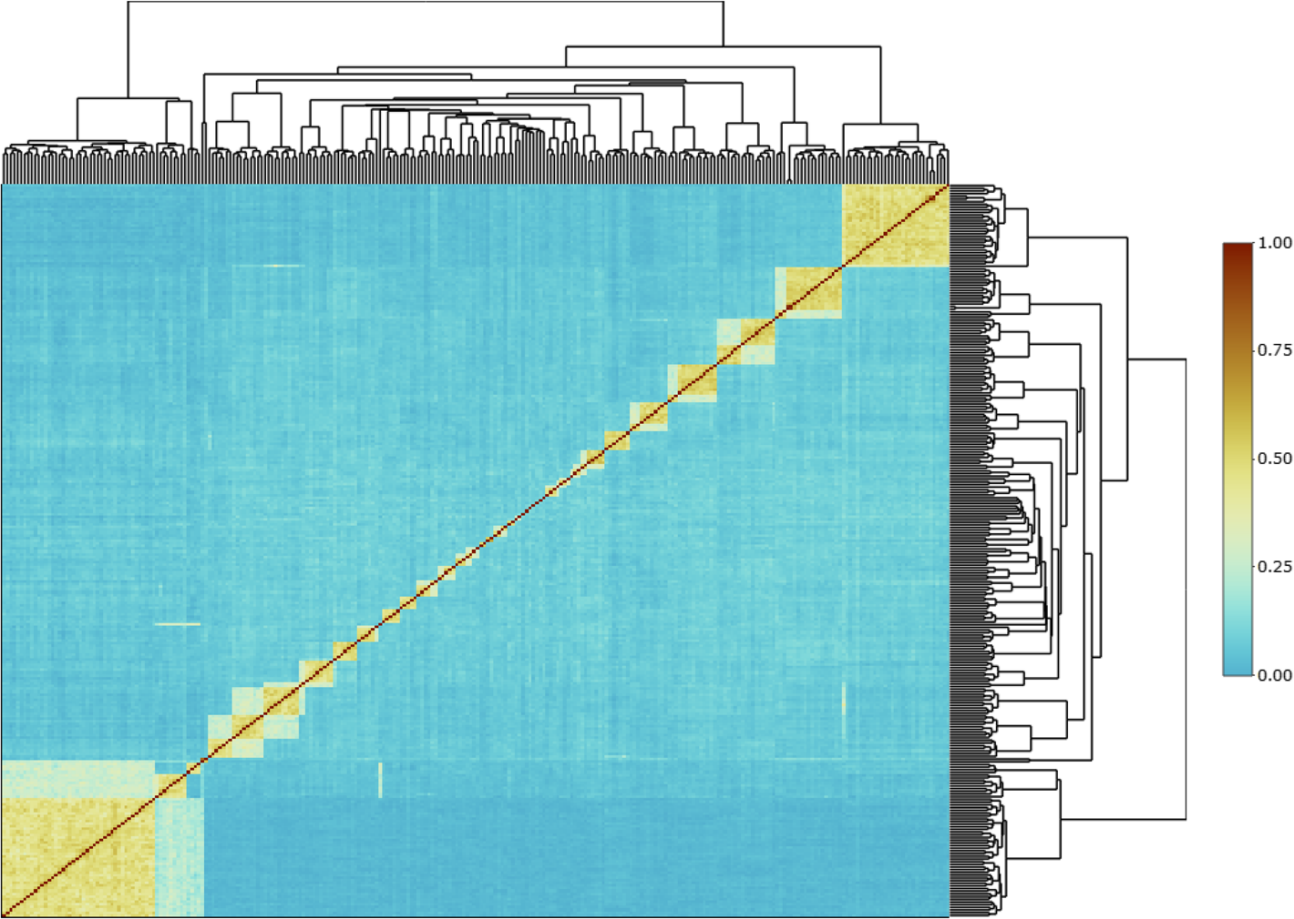
Heat-map with dendrogram clustered from a genomic relationship matrix (GRM) of commercial, communally spawned black tiger shrimp progeny. The pixel colouring denotes proportion of genomic relationship between two individuals with 0 = no relationship and 1 = identical. Plotted with R package *heatmaply*

To further explore the utility of the 4194 DArTcap SNP panel, genomic relationship values were calculated for an additional independent set of G2 samples (*n* = 272) that were produced from novel Northern Territory sourced broodstock (i.e. not genotyped with either DArTseq or DArTcap). Utilising the GRM values of progeny alone, in the absence of parental genotypes, it is possible to clearly obtain sib-ship information (block structure in Fig. 4), including delineating full-sib and half-sib relationships. Furthermore, when compared to routine pedigree based relationship matrixes, it is possible, by assessing the values off the diagonal of the heat-map (within and outside blocks), to identify cryptic relatedness between otherwise traditionally unrelated individuals (Fig. 4).

#### Population segregation

**Fig. 5:**
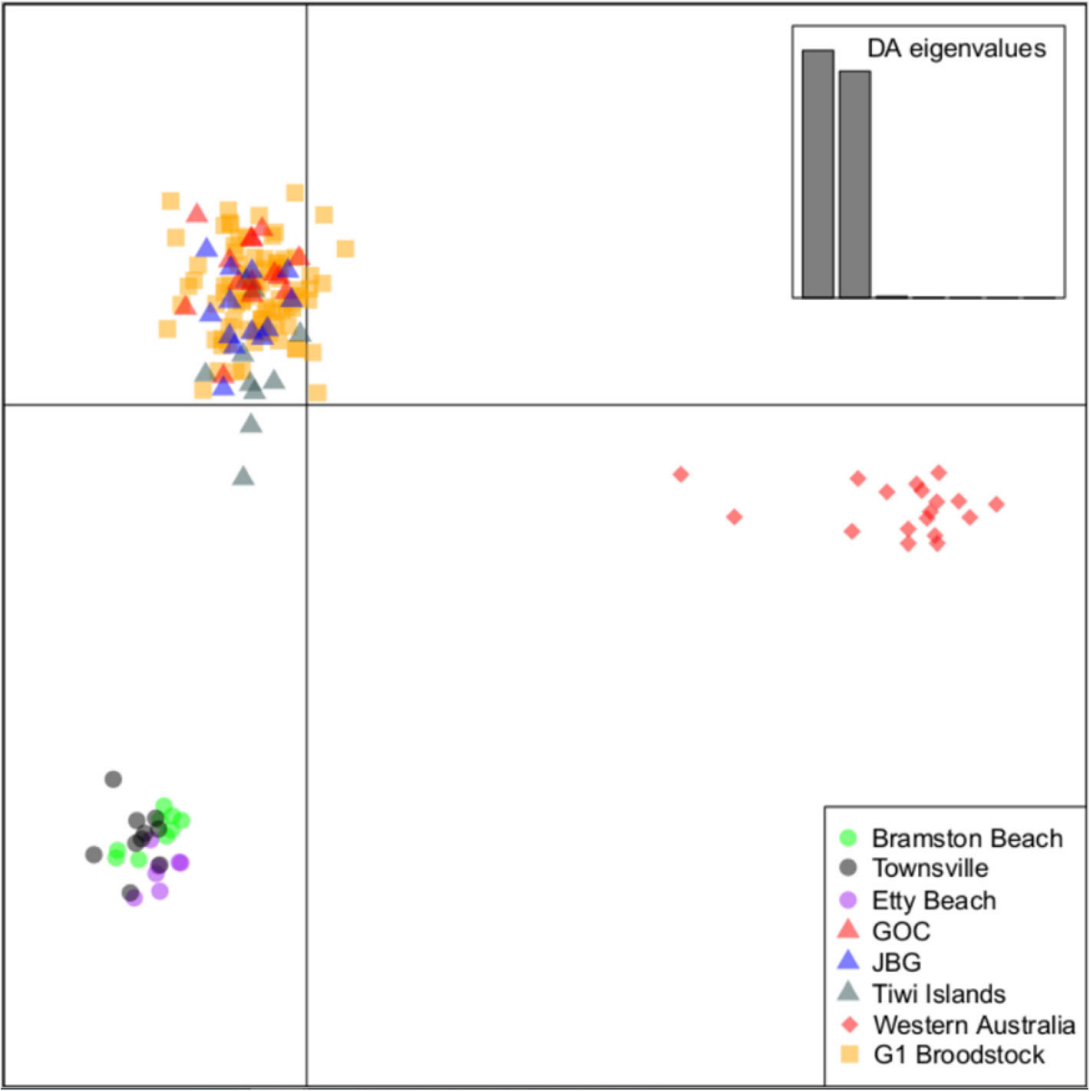
Clustering of 168 wild-sourced samples based upon genetic similarity shown through discriminant analysis of principle components (DAPC). PC1 and PC2 are shown on the x and y axis respectively. PC1 explains 52.8% and PC2 explains 44.3% of the variation

The ability of the 4194 DArTcap SNP array to segregate samples obtained from different populations across the Australian distribution of *P. monodon* was assessed by comparing these individuals through discriminant analysis of principle components (DAPC). By comparing the first two principle components (with PC1 explaining 52.8% and PC2 explaining 44.3% of the variation), three distinct clusters of individuals are clearly identified (Fig. 5). Specifically, samples obtained from within East Australia Coast, Northern Territory and Western Australia form their own clusters. The G1 broodstock and G2 progeny also cluster with their source population (Northern Territory) showing no clear separation as a result of breeding practices (Supplementary Figure 2). The associated regional structure is consistent with previously identified population structure [22].

## Discussion

Black tiger shrimp aquaculture is of substantial economic value globally and is forecast to expand rapidly in Australia with consistently increasing demand in domestic and international markets. However, the industry as a whole has lacked a viable low-cost genotyping assay capable of being used for advanced selective breeding programs, including genomic selection. In this study, we undertook *de-novo* SNP discovery, marker quality control and filtering, before selecting and successfully validating a custom DArTcap genotyping assay containing 4194 SNPs across Australian populations of *P. monodon*. Access to such assays is highly sought after within *P. monodon* breeding programs, particularly to facilitate the transition to genomic selection.

### DArTcap assay development

#### SNP discovery

In this study, we obtained 31,262 SNPs (24,683 unique RAD-tags) by employing *de-novo* SNP discovery (DArTseq) genotyping of 650 individuals collected from across the natural range of Australian *P. monodon* [22]. The number of markers (and RAD-tags) obtained in this study are closely comparable to studies using 2b-RAD [33] and SLAF-seq [20] in white leg shrimp (*Litopenaeus vannamei*) with 25,140 SNPs, and 23,049 SNPs obtained previously. A recent study using SLAF-seq for *P. monodon* obtained a lower total number of markers (6821) than this study, however, these were reported after completion of filtering (markers retained based on Parent read depth (10–200), 90% call rate, MI > 0.01) and as such is not directly comparable. Furthermore, these markers were utilised for linkage map construction, rather than with the intent of inclusion in a genotyping assay, and as such a single large family (98 G2 progeny, G0 stock derived from Mozambique) was used in SNP discovery [34] rather than employing a diverse discovery population. Previously, Baranski [28] utilised transcriptome sequencing (RNA-seq) of *P. monodon* obtained from four locations around the coast of India, to discover 473,620 SNPs. Baranski [28] proceeded to produce a custom solid-state 6 K Illumina iSelect genotyping assay, and a subsequent genetic linkage map with the completed assay, which has been further utilised in GWAS and QTL studies [35]. In this study we aimed to produce a low-cost genotyping assay that relied upon genotype-by-sequencing (requiring a restriction enzyme complexity reduction), and therefore were not able to directly integrate existing SNP resources from Baranski [28]. Furthermore with the intended focus on utilisation of the DArTcap assay within the Australian industry, it was important to derive SNPs from Australian stock, to ensure marker ‘informativeness’ was maximised.

#### Determining SNP density required for GRM analysis

Since the initial shift from traditional co-dominant markers (e.g. microsatellites) to SNPs, there has been a focus on increasing the density of SNPs included in assays for many commercially important species. Particularly with the SNP discovery through whole genome re-sequencing approaches, and improvement of genotyping techniques, commercially accessible assays have grown from thousands of markers, to hundreds of thousands markers for some species [e.g. salmon 130 K [36], catfish 250 K [37] and 690 K [38], common carp 250 K [39]]. While this is useful for some applications (e.g. genome wide association studies), in many situations, genotyping at this marker density is not economically feasible, or required to complete the desired analysis [40, 41]. For a number of species with a closed nucleus breeding design, lower density SNPs assays with a few thousand markers are now being developed for routine breeding applications for many species [e.g. cattle [42], chickens [43] and salmon [40]].

In this study, for *P. monodon* we identified that 4000 markers were sufficient for the design of the DArTcap array to ensure high concordance was obtained for GRM calculations when compared to those derived from all available filtered DArTseq markers (7542 SNPs; Fig. 2). For use of this assay in future projects we desired a correlation of 98%, however, depending on the end use application and required genotyping costs, the marker density could be reduced to between 2000 and 2500 markers while still achieving approximately 95% correlation to the 7542 DArTseq SNPs. Reducing marker densities further would result in the rapid decay of GRM correlations and is not advised for using in advanced genomic based breeding.

Similar studies have previously been completed for *L. vannamei*, and indicated 3000 SNPs were appropriate for GRM calculations [44]. Likewise, Wang [45] found 3200 SNPs for *L. vannamei* would be sufficient for accurate GRM calculations when assessing an independent commercial population. While the genome sizes of *P. monodon* and *L. vannamei* are similar, and karyotypes are identical [2.2 Gb vs 1.7 Gb; 44 chromosomes; Guppy [46]], the lower required number of markers for GRMs in *L. vannamei* may be due to both studies utilising samples from established breeding programs (many generations removed from wild) with large full-sib/half-sib family structures rather than wild individuals from two distinct regions in this study [47]. Effectively, a large full-sib/half-sib family dataset structure requires fewer markers to completely tag each segregating portion of the genome, and as such results in lower variation in GRM estimates at lower marker densities [48–50]. In future studies it would be valuable to reassess the required density of markers for *P. monodon* stock after multiple generations of domestication. Species with smaller and/or less complex (less polymorphic/repetitive elements) genomes or low effective population sizes may be able to use smaller arrays and should undertake a similar approach when designing an assay.

#### SNP linkage disequilibrium

While a small number of marker pairs were observed to have linkage disequilibrium (LD) greater than 0.8, only one of these pairs was regarded as highly linked in both Northern Territory and East Australian Coast regions. Given the genetic distinction between regions (Fig. 5), it is expected that the patterns of LD across individuals from each region will be different as well, and markers in low LD in one population may be unlinked (or show a varied level of linkage) in another [51, 52]. As such, these markers were not removed from the assay.

Furthermore, unlike livestock species which have low effective populations sizes (with highly similar organisation of genomes between individuals), the majority of commercial *P. monodon* cultured in Australia are not yet distinctly separated from wild stock (Supp. Figure 2), and will exhibit a large degree of varied genome arrangement across various individuals. Further investigation is warranted, however, as patterns of LD, both across regions and within regions, will impact the ability to translate genomic selection models (both in GRMs and estimating SNP effects) across to commercial stocks derived from different regions [41, 53, 54].

While not available at the time of SNP selection, an early draft assembly of the *P. monodon* genome has since been made available [55]. Mapping markers to this assembly could be an additional approach used to filter out loci by genome position; however, the assembly contains over four million contigs and as such, currently has insufficient contiguity to be informative for this approach. Further work is underway to improve the *P. monodon* genome assembly, and the distribution of SNPs in this assay should be assessed further when this is available.

### Assay performance

The conversion rate of the selected probes into a successful assay was high, with 99% of the targeted 4236 markers returned. Furthermore the genotyping call rate across the individuals resubmitted for DArTcap genotyping was high (92.40% ± 2.75% SD), and has remained high in subsequent genotyping of independent samples from additional Australian populations (Western Australia) and commercial stock (93.50% ± 3.90% SD). Furthermore, while the average call rate obtained across all individuals (93.05% ± 3.51% SD) in this study with DArTcap genotyping is marginally lower than those achieved with solid state genotyping [e.g. 98.92%, Illumina Infinium ShrimpLD-24; Jones [56]], it is markedly higher than call rates achieved through traditional de novo GBS approaches (e.g. 86% in raw DArTseq data for this study). The improvement in data quality obtained with DArTcap genotyping over de novo GBS is further evident after the removal of low confidence genotype calls from the DArTseq dataset as the call rate decreased to 66%. Similarly, while the average MAF of the DArTcap SNPs (0.23 ± 0.15) was lower than reported by Jones [56] (0.37), the DArTcap assay holds sufficient ‘informativeness’ for routine applications including assigning parentage (Table 2) and assessing genomic relatedness (GRM; Fig. 4) or population structure (Fig. 5).

A secondary SNP (additional non-targeted polymorphism with the target sequence) was obtained for a number of the probes and for routine application should be removed (as these are in complete linkage with target SNP); however they may represent additional valuable information if combined into RAD-tag haplotypes [57], or when the assay is applied to populations where the allele frequency of the target SNP is not as informative as the original tested populations (e.g. rare or fixed). Given the global value of the *P. monodon* industry it would be of interest to further assess the utility of this assay across potentially divergent international populations and commercial stocks.

The sequencing effort applied to each sample was markedly reduced for each individual (from 2.5 million to 670,610 reads per individual total) in DArTcap over DArTseq; however the average read depth obtained for each genotype call was higher (80.03 reads in DArTcap vs 17.01 reads DArTSeq), substantially increasing the confidence and accuracy of genotype calls. Furthermore, given sequencing costs are the most substantial cost associated with GBS, being able to reduce the total allocation required to obtain robust data is critical for routine applications involving the genotyping of thousands of individuals annually as required by breeding programs or when undertaking genome-wide association studies. Through utilising DArTcap genotyping over DArTseq genotyping, three times the total number of individuals can be assessed for an equivalent fixed financial investment (accounting for fixed per sample costs such as DNA extraction, service provider labour).

### Assay utilisation

#### Parentage assignment

The ability to undertake parentage assignment remains a fundamental requirement for the vast majority of commercial farms and research end users. In many aquaculture production systems, including the *P. monodon* industry, progeny are produced from mass spawning events of multiple broodstock, with progeny of many families being reared communally from fertilisation onwards. Quite often it is not possible to manually tag or separate family lines. As a result, genotyping progeny and broodstock, and assigning parentage through molecular means is essential as it enables the recovery of pedigree information and ensures breeding programs are managed effectively [27, 58].

The parentage assignment success rate obtained in this study when using the 4194 DArTcap was high (up to 98.61%), and further validates the utility of this assay more broadly. False assignment was not observed within any parental assignment analysis over the three datasets and various genotyping error rates, and would be highly unusual given the statistical approach employed in Colony [30]. Furthermore, false exclusions rates were low across all analyses (with the exception of DArTcap parentage run at 1% error) with only a single individual performing consistently poorly irrespective of SNP set or other analysis variables. With the 4194 DArTcap SNP sets, the rate of false exclusions were sensitive to the estimated genotype error rate included in the analysis, however, this is well known [59] and can be easily accommodated for in analysis.

Unlike in both DArTseq datasets, individual Mendelian inheritance errors (single genotype calls) were not silenced in the DArTcap dataset, and will have contributed to a large degree of the variation in exclusion rates seen between assays at each estimated genotyping error rate [60]. This approach was taken for validation purposes, as we wanted to only utilise prior parentage knowledge to valid assignments, rather than to assist in the analysis itself (e.g. including a single parent known). This provided the best equivalent to circumstances seen in routine commercial situations where no parental data can be linked in advance.

Depending on marker polymorphism and the relatedness of individuals, small arrays of 80–200 SNPs are often sufficient for parentage assignment [60, 61]. In previous studies using solid state technology (Sequenom) rather than GBS, Sellars [26] found similar assignment success (> 95%) to those achieved in this study are possible by using assays including 122 SNPs on eighth generation domesticated *P. monodon* stock. The parentage assignment success of the 122 SNPs assay provided by Sellars [26] was higher when compared to previous approaches with 12 or 13 microsatellites [62, 63]. Further investigation of specific parentage subsets within the DArTcap assay should be explored to allow substantial reduction in time and computational requirement involved in assignment. To date, no direct studies of parentage assignment using GBS for pooled samples are available, but should be explored to further reduce genotyping expenses [46, 64].

#### Genomic relationship calculations

While comparisons of genomic relationships between the two assay types have been discussed, for the purposes of validating the assay we further tested the ability of the GRMs calculated from the DArTcap array across an independent communally reared commercial progeny cohort. When visualising the genomic relationship matrices with a dendrogram clustered heat map (Fig. 4), it is evident that full-sib and half-sib relationships can be separated into their respective family groupings, even in the absence of reference to any parental genotype or manual pedigree information. Furthermore a range of cryptic relationships are evident in the group of individuals tested, whereby individuals share either higher or lower relatedness values than expected in the traditional discrete pedigree relationships (i.e. 0 = unrelated, 0.25 = half-sib, 0.5 = full sib).

For genomic selection applications, it is not necessary in all circumstances to be able to obtain full parentage information (i.e. parent – progeny relationship), but instead determine an accurate estimate of the relatedness of individuals included in both the testing and training populations datasets [49]. Under circumstances where the true relationship between two individuals is inaccurate, their relative value or merit in breeding programs (EBV or GEBV) can be also incorrectly estimated [49]. The increase in selection accuracy by utilising GRMs has been well studied in terrestrial agriculture species [65] and has seen increasing attention in aquaculture breeding programs [40, 66–68]. When compared to mass selection approaches that are currently available for the black tiger shrimp aquaculture industry, utilising GRMs simultaneously allows increased accuracy of selection [69], and further mitigation of inbreeding [e.g. optimised contribution selection [70];].

#### Population segregation with DArTcap

The ability to identify cryptic genetic sub-structuring within populations or groups can be useful to not only identify unique genetic stocks for founder selection [71], but also to trace captive lineages back to wild source populations [72], or identify escapees from aquaculture facilities [73].

Currently translocation of *P. monodon* broodstock occurs under permits between the Northern Territory and the East Australia Coast. Northern Territory broodstock in particular are captured from wild fisheries, and spawned in contained facilities in East Australia Coast region. The DArTcap assay was successfully able to reassign Northern Territory, East Australia Coast and Western Australia samples to their true population of origin (Fig. 5). If an escape event was suspected, this assay could be applied successfully to trace sampled individuals (broodstock or progeny) back to their source population (Sup Figure 2) This approach has been explored extensively in salmonoid aquaculture [74, 75], as well as in other species (e.g. turbot [76]).

### Considerations for future filtering of GBS and SNP selection GBS assays

While genotype-by-sequencing approaches are promising and are being applied across multiple species [1, 9, 46], there are a number of considerations that need to be addressed. Compared to solid state genotyping on fixed arrays (i.e. Illumina, Affymetrix), GBS approaches usually yield lower genotyping accuracies due to the nature of low read coverage in some SNPs. Filtering tailored to specific datasets can remove genotype calls with lower accuracies which in turn increase the accuracy and quality of the dataset as a whole, however, overly aggressive filtering can also remove large extents of data, much of which is of sufficient quality for downstream analysis [9]. Insufficient filtering thresholds, or absence of specific criteria themselves (e.G. *minimum* read depth) can result in spurious genotypes persisting in the dataset and being used in subsequent downstream analyses [77–79].

During the design of the 4 k DARTcap array, we aimed to use balanced SNP filtering thresholds that retained as much data as possible, while removing the SNPs and genotype calls for which we had low confidence. A number of major genotyping performance metrics were explored including call rate, minor allele frequency, Mendelian error rates and Hardy-Weinberg deviations. It is interesting to note that a number of these metrics are intrinsically linked [e.g. call rate and read depth, read depth and MI error, repeatability and read depth [9];]. In addition, when transitioning from DArTseq to DArTcap procedures, a number of metrics (Call rate, read depth, reduced MI errors) were expected to improve dramatically [13, 16].

As such we placed the highest emphasis on marker minor allele frequency and call rate to ensure the allele frequencies remain high enough for use across multiple generations of selective breeding, and the markers were present in as many genotyped individuals as possible (avoiding population specific markers and those that were difficult to sequence). With reduced error in mind, markers showing on average higher read depths and higher repeatability of genotype calls (indicating consistent sequencing both between and within individuals) were preferentially ranked well. Furthermore, SNPs with low levels of MI error and small deviations from HWE were expected to improve with increased sequencing depth of DArTcap genotyping, and comparatively were not ranked down aggressively, instead the SNPs representing the majority of MI errors (> 9%) and significant deviations from HWE in both populations were removed earlier.

Markers with excessive read depth (e.g. > 200 reads/ genotype) should also be avoided, as this may indicate two markers erroneously being called together from paralogs (over-clustered), or located in highly repetitive regions [41, 77]. Filtering these markers was overlooked during the assay design phase of this study. While we removed secondary loci from each RAD-tag, it is also advisable to avoid highly polymorphic RAD-tags as the clustering undertaken during the bioinformatics processes involved in SNP calling becomes inherently more complex, and can lead to over-splitting of markers due incorrect phasing of alleles [41, 79]. Lastly, when selecting RAD-tags consideration should be taken to ensure the target SNP is not located near the fragment end to avoid issues with tag binding. Similarly, selection criteria for inclusion markers within other genotyping technologies should be considered where possible. For instance, SNP position is critical in Illumina probe design, and may hinder the transferability of the markers developed in this study to other genotyping assays.

## Conclusions

A method to routinely genotype thousands of individuals is required to undertake advanced selective breeding in black tiger shrimp. This study described the development and validation of a crucial genotyping resource, which by utilising target capture genotype by sequencing (DArTcap) can for the first time be cost-effectively utilised in routinely commercial breeding (< $15 AUD per sample). This assay containing 4194 SNPs, simultaneously provides the ability reconstruct the pedigree of communally reared families, while also being able to accurately calculate the cryptic genomic relationships between individuals. Furthermore, this assay will facilitate genome-wide association studies, linkage mapping, and unlocks the ability to undertake genomic selection black tiger shrimp.

## Methods

### Sample collection and DNA isolation

To develop informative genotyping assays, it is necessary to compile as diverse discovery population as practically possible while encapsulating the range of individuals to be utilized within the industry breeding programs. As such, samples were collected from a number of sources across the natural range of Australian *P. monodon* (*n* = 264; Supplementary Figure 1) and from 1st and 2nd generation farm stock (*n* = 165 and *n* = 231 respectively). Given the aims and design of this study, a priori sample size calculations were not appropriate. Farm stock were provided for sampling by Seafarms Group Ltd. as part of routine operational practises, while individuals from natural populations were obtained from CSIRO fishery surveys and were collected within the guidelines of appropriate permits for each location. All individuals were euthanized upon collection through immersion in an ice-slurry. Pleopod tissue or whole post larvae were collected and stored in ethanol or RNA-later at − 20 °C until extraction. Genomic DNA was extracted either following the CTAB extraction protocol or MagJET Genomic DNA Kit ([80] and ThermoFisher Scientific). Genomic DNA was purified using Sephadex G-50 (GE Healthcare Life Sciences 2000) and visualised on 0.8% agarose gel to ensure quality and quantity were sufficient.

### Genome-wide SNP discovery

In the absence of existing published GBS datasets for *P. monodon*, a reduced-representation genome by sequencing (GBS) approach, DArTseq, was employed for SNP discovery through a service provider, Diversity Arrays Technologies (DArT [4];). High quality DNA was provided to DArT to identify SNP markers through a restriction digest reduced-representation based sequencing strategy as described in [4, 81]. Briefly, DNA from 660 shrimp underwent a restriction digest using *Pstl* and *Hpall* restriction enzymes and unique proprietary barcodes for each sample were ligated to size-selected DNA. Samples that demonstrated non-uniform digestion patterns were excluded from library preparation (*n* = 10). To allow a measure of technical repeatability in genotype calls and library preparation, 147 random replicates (23%) were included within the library preparation process. Equimolar amounts of barcoded samples were pooled, with 94 samples forming a single pool, before sequencing cluster preparation on the Illumina c-Bot bridge PCR system. Libraries were sequenced on three flow cell lanes on an Illumina HiSeq2500 to provide an average of 2.5 million raw reads per individual.

Reads with low sequence quality scores (Q < 25) were eliminated and SNP calling was completed using the KDcompute pipeline (DArT [81];). Following this, both monomorphic loci and sequences associated with species other than *P. monodon* (human, bacterial etc.) were excluded from the data set.

### Marker quality control

To ensure only high quality markers were included in the final target capture genotyping assay, multiple steps of filtering were applied to the dataset before the remaining SNPs were ranked on quality and level of polymorphism prior to final selection. Custom python scripts were developed to efficiently handle the datasets provided by DArT (github.com/esteinig/dartQC) and undertake preliminary SNP filtering for quality. Briefly, genotype calls were silenced on the basis of low cumulative raw read depth (i.e. with sum of reads for both alleles less than five), before SNPs with minor allele frequency (MAF) less than 0.02, call rate less than 50% or less than 90% repeatability were removed. Next, SNP redundancy filtering was undertaken, whereby sequence clusters (RAD-tag or clones) were identified using the CD-HIT clustering algorithm at 95% identity [82], and then the SNP with the highest MAF within each sequence cluster was retained. This redundancy filtering was undertaken to ensure that overrepresentation of specific areas of the genome did not occur, as this may introduce bias into future genomic analyses.

Hardy Weinberg Equilibrium (HWE) deviations were calculated within PLINK [83] utilizing discrete datasets for the East Coast (*n* = 132) and Northern Territory stocks (*n* = 125) (Table 1). SNPs identified to significantly deviate (*p* < 0.0001) from HWE in both populations were removed. Known parental trio relationships (81, progeny-dam-sire) were utilised to identify SNPs that displayed Mendelian Inheritance (MI) errors using PLINK [83]. MI errors were investigated on an individual SNP call basis as these could be due to incorrect genotype calls from sequencing error, or insufficient read depth [84]. SNPs with high levels of aberrant MI errors (> 9%) not associated with sequence coverage errors were removed.

### SNP selection and assay design

To determine the number of markers required to accurately obtain GRMs in *P. monodon*, calculations were undertaken using a similar approach to Rolf [42]. GRMs calculated from increasing densities of markers were compared to a GRM estimated from all 7542 SNPs (**G**). The programs R *v3.4.1* and PLINK *v1.9* [83] were used to create randomly selected marker subsets (*n* = 100, 200, 500, 1000, 1500, 2500, 3000, 3500, 4000, 4500, 5000, 5500, 6000, 6500, 7000), with replacement, from the pool of 7542 SNPs available. For each marker subset (*n)*, 50 random replicates (*i*) were generated and a GRM (**G**_***ni***_) was estimated using GCTA *v1.91.7b* [85]. Correlations were then drawn between the pairwise relatedness of 650 individuals (Table 1) produced in each **G**_***ni***_ GRM and the corresponding relatedness values of **G**. Average pairwise relatedness values across the 50 replicates for each marker subset were calculated before correlation plots were produced using the R package *ggplot2* [86]. Such GRM analysis (detailed below) indicated that a panel of 4000 SNPs was able to produce a GRM with an *r*^*2*^ correlation of 0.98 to the full 7542 array. Therefore, this defined the target size of the DArTcap sub-array to be developed.

To prioritise the selection of the highest quality SNPs in the final assay, a custom quality score (*QC score*) was developed to rank each SNP by a range of SNP quality metrics (Eq. 1). Metrics included were, call rate (*CR*) which ranged from 0 to 1 with a weighting of 1.4; minor allele frequency (*MAF*) ranging from 0 to 0.5 with a weighting of 2.4; average repeatability (*Rep_Avg*) ranging from 0 to 1; standardised read depth (*RD*) whereby the average read depth of the SNP was divided by the largest read depth observed to give a proportion between 0 and 1; Hardy Weinberg Equilibrium deviation (*HWE*) as a percentage; and Mendelian inheritance error rate (*MI*).

1 $$ QC\  score=\left( CR\times 1.4\right)+\left( MAF\times 2.4\right)+ Rep\_ Avg+ RD-\frac{(HWE)}{10}- MI $$

A total of 4236 highest ranked QC score SNPs were selected for the 4 K genotyping assay allowing for some redundancy for marker drop out (cross probe affinity, probe failure) during probe synthesis. Furthermore, a GRM was calculated using the 4236 subset of markers and compared to the full 7542 markers to ensure concordance was maintained. Selected sequences were provided to DArT, and DArTcap probes synthesized (MYbaits®, MYcroarray®) for testing.

### Linkage disequilibrium

To approximate the distribution and independence of markers across the genome, linkage disequilibrium (LD) was calculated across the complete datasets of 7542 SNPs and 4236 DArTseq SNPs with all available samples in PLINK [83]. Similarly, population specific LD was calculated with samples from the Northern Territory (*n* = 125) and East Australian Coast (*n* = 132) regions independently. Pairwise LD values between loci greater than > 0.2 were reported, and then compared between Northern Territory and East Australian Coast regions to identify if any markers were consistently in complete (*r*^*2*^ = 1) or high LD (*r*^*2*^ > 0.8).

### Assay validation

Validation of the DArTcap probes involved the re-genotyping of a subset of 251 individuals from the discovery population (Table 1). DArTcap follows similar methodology to DArTseq, however, it involves an additional magnetic bead hybridization step (Dynabeads, MyOne) that utilises the DArTcap probes to capture and enrich the 4236 target SNP sequences before being put forward for sequencing. Sequencing of the targeted (DArTcap) library and preliminary sequence data quality control was identical to the DArTseq procedure described above. A minimum of 8% technical replicates (i.e. 8 samples per 94 well plate) were included to provide a measure of SNP repeatability.

In addition to sequencing a representation of the DArTseq discovery population, two groups of additional novel individuals (19 individuals from Nickol Bay, Western Australia, and 282 additional commercial progeny; Table 1) were also included in the DArTcap sequencing effort as independent datasets for validation and analysis. In order to evaluate the quality and integrity of the DArTcap assay, comparisons were drawn between SNP metrics (i.e. call rate, read depth, Mendelian inheritance errors) produced by the two GBS methods (DArTseq and DArTcap). In addition, parentage analysis, GRM calculations and sample relatedness were also assessed with the 7542 DArTseq SNP dataset, the 4236 DArTseq SNP dataset and the final 4194 DArTcap SNP dataset to validate the utility of the finalized assay.

### Parentage assignment

As parentage assignment is a fundamental requirement for many genotyping assays, power to assign parentage was tested across three SNP datasets (the 75,442 filtered DArTseq SNPs, the 4236 DArTseq SNPs selected for assay design and the 4194 DArTcap SNPs). A total of 46 progeny and 56 broodstock with known parent-progeny relationships were available for genotyping on both the DArTseq and DArTcap platforms. A number of additional broodstock with known parent-progeny relationships had insufficient DNA when undertaking DArTcap genotyping and were excluded. For these 72 family pairwise relationships, parentage assignment was undertaken in the program Colony [30]. Repeated analyses with estimated genotyping error rates ranging from strict (1%), to moderate (5%), to conservative (10%) were completed to account for undefined genotyping error rates across the three SNP datasets [87]. Since the progeny were the first generation of wild broodstock pairings, inbreeding was not included. Prior sib-ship assumptions were excluded allowing for highly skewed family sizes commonly observed in mass-spawning aquaculture systems [58]. Likewise maternal and paternal polygamy were allowed to account for potential of half-sib breeding designs that utilise artificial insemination. All analyses were completed with the ‘long run’, ‘high precision’ and ‘full-likelihood’ options within Colony. The two types of parentage assignment errors; a) incorrect exclusion of a true parent, and b) assignment of an untrue parent, were determined for each assignment analysis.

### Calculating relatedness and identifying genetic structure

To confirm the estimates of relatedness obtained from DArTcap genotyping (4129 SNPs) were concordant with those of the selected 4236 DArTseq markers, a GRM was calculated in GCTA v1.91.7b for both datasets and all common individuals (*n* = 195) in the two datasets were compared..

To provide a complementary approach to visually assess the utility of GRMs derived from DArTcap genotyping, GRMs and dendrograms were calculated for an independent set of G2 samples (*n* = 272; Table 1) and then plotted as a heat-map in R with the package ‘*heatmaply*’ [88].

To confirm the DArTcap assay retained sufficient informativeness to distinguish between individuals from distinct populations, we completed discriminant analysis of principal components (DAPC) using the R package *adegenet* [89]. We completed this analysis with three subsets of the 4194 DArTcap SNP dataset. Firstly, all available individuals (*n* = 418) secondly retaining only wild sourced broodstock and wild samples and excluding all G2 individuals (*n* = 168) to ensure closely related individuals were not influencing the analysis, and lastly with only an independent set of G2 samples (*n* = 272) to assess the ability to discriminate between family lines. Concordance of sample placement within source populations was assessed.

## Supplementary information

**Additional file 1: Supplementary Figure 1.***Penaeus monodon* distribution across Australia (light grey), approximate locations of current pond based farming operations (dark grey), and location of samples included in DARTcap ‘discovery’ populations (1 – Joseph Bonaparte Bay (*n* = 34), 2 – Tiwi Islands (*n* = 56), 3 – Gulf of Carpentaria (*n* = 43), 4 – Bramston Beach (*n* = 60), 5 – Etty Bay (*n* = 50), 6 – Townsville (*n* = 22) and 7 – Commercial Farm site (*n* = 394). **Supplementary Figure 2**. Clustering of samples based upon genetic similarity shown through discriminant analysis of principle components (DAPC). PC1 and PC2 are shown on the x and y axis respectively. (A) Including all samples (*n* = 418). (B) Including only second generation (G2) individuals (*n* = 272) obtained from routine commercial spawning.

## Data Availability

The DArTseq and DArTcap datasets files generated during the current study, including read counts and genotypes, are available on the DRYAD data repository (10.5061/dryad.qz612jmc8).

## Abbreviations

AFLPs: Amplified fragment length polymorphisms
CTAB: Cetyl trimethylammonium bromide
DAPC: Discriminant analysis of principle components
DArT: Diversity Arrays Technologies
DNA: Deoxyribonucleic acid
EBV: Estimated breeding value
G1/G2: Generation 1 and 2
Gb: Gigabase
GBS: Genotype-by-sequencing
GEBV: Genomic estimated breeding value
GRM: Genomic relationship matrix
GS: Genomic selection
GWAS: Genome wide association studies
HWE: Hardy-Weinberg equilibrium
LD: Linkage disequilibrium
MAF: Minor allele frequency
MI: Mendialian inheritance
QC: Quality control
QTL: Quantitative trait locus
RAD-Seq: Restriction site Associated DNA Sequencing
SD: Standard deviation
SNP: Single nucleotide polymorphism
PC: Principle components
