# Supplementary material for: Development and validation of a RAD-Seq target-capture based genotyping assay for routine application in advanced black tiger shrimp (Penaeus monodon) breeding programs
Source: BMC Genomics. 2020 Aug 5;21:541. doi: 10.1186/s12864-020-06960-w (PMC7430818; doi:10.1186/s12864-020-06960-w)
Supplement: Supplementary file 1 — Additional file 1: Supplementary Figure 1.Penaeus monodon distribution across Australia (light grey), approximate locations of current pond based farming operations (dark grey), and location of samples included in DARTcap ‘discovery’ populations (1 – Joseph Bonaparte Bay (n = 34), 2 – Tiwi Islands (n = 56), 3 – Gulf of Carpentaria (n = 43), 4 – Bramston Beach (n = 60), 5 – Etty Bay (n = 50), 6 – Townsville (n = 22) and 7 – Commercial Farm site (n = 394). Supplementary Figure 2. Clustering of samples based upon genetic similarity shown through discriminant analysis of principle components (DAPC). PC1 and PC2 are shown on the x and y axis respectively. (A) Including all samples (n = 418). (B) Including only second generation (G2) individuals (n = 272) obtained from routine commercial spawning. [file 12864_2020_6960_MOESM1_ESM.docx]

**Supplementary Figures**


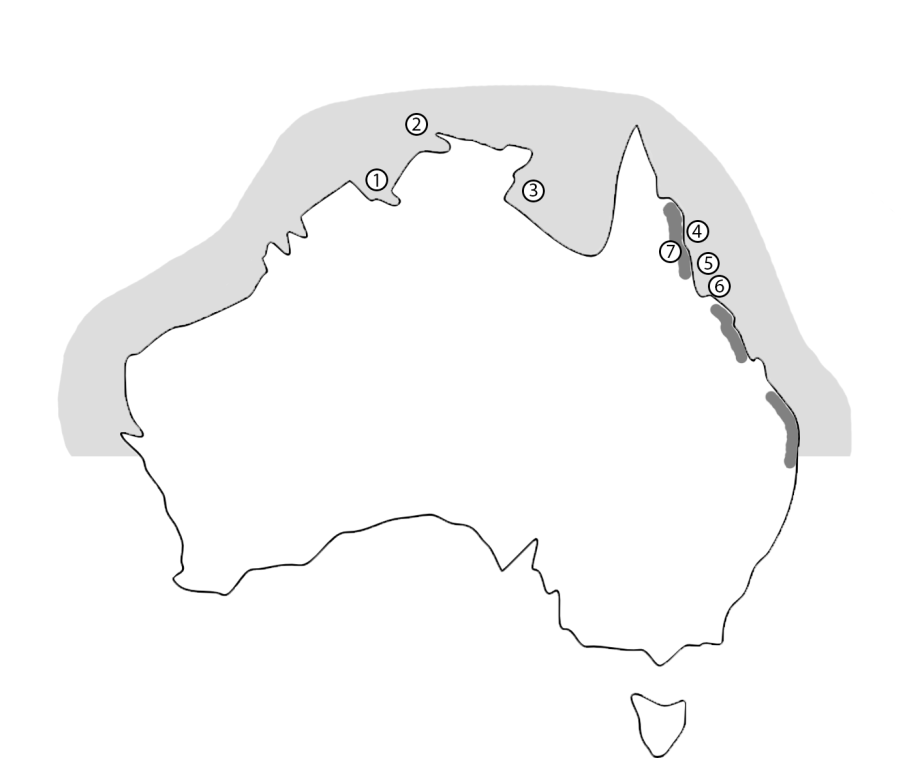


**Supplementary Figure 1.** *Penaeus monodon* distribution across Australia (light grey), approximate locations of current pond based farming operations (dark grey), and location of samples included in DARTcap ‘discovery’ populations (1 – Joseph Bonaparte Bay (n = 34), 2 – Tiwi Islands (n = 56), 3 – Gulf of Carpentaria (n = 43), 4 – Bramston Beach (n = 60), 5 – Etty Bay (n = 50), 6 – Townsville (n = 22) and 7 – Commercial Farm site (n = 394).

**
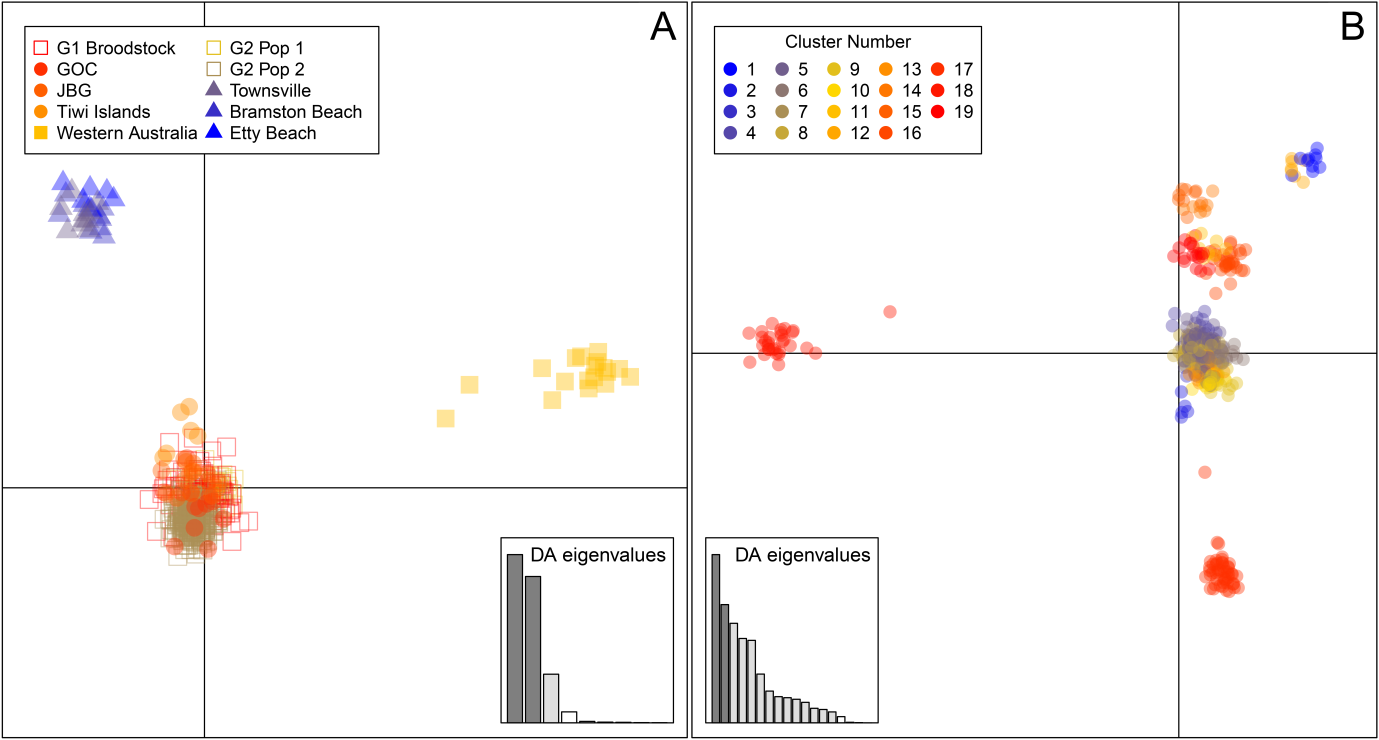
**

**Supplementary Figure 2**. Clustering of samples based upon genetic similarity shown through discriminant analysis of principle components (DAPC). PC1 and PC2 are shown on the x and y axis respectively. (A) Including all samples (n = 418). (B) Including only second generation (G2) individuals (n = 272) obtained from routine commercial spawning.
